# Australian Honeypot Ant (*Camponotus inflatus*) Honey—A Comprehensive Analysis of the Physiochemical Characteristics, Bioactivity, and HPTLC Profile of a Traditional Indigenous Australian Food

**DOI:** 10.3390/molecules27072154

**Published:** 2022-03-27

**Authors:** Md Khairul Islam, Ivan Lozada Lawag, Tomislav Sostaric, Edie Ulrich, Danny Ulrich, Terrence Dewar, Lee Yong Lim, Cornelia Locher

**Affiliations:** 1 Cooperative Research Centre for Honey Bee Products Limited (CRC HBP), University of Western Australia, Perth, WA 6009, Australia; mdkhairul.islam@research.uwa.edu.au (M.K.I.); ivan.lawag@research.uwa.edu.au (I.L.L.); 2 Division of Pharmacy, School of Allied Health, University of Western Australia, Crawley, WA 6009, Australia; tom@chromatechscientific.com (T.S.); lee.lim@uwa.edu.au (L.Y.L.); 3 15 Pira Avenue, Kalgoorlie, WA 6430, Australia; edieulrich@gmail.com (E.U.); dannyulrich1844@gmail.com (D.U.); 4 Australian Biome, 11 Oxted Place, Morley, WA 6062, Australia; terrence.dewar@australianbiomeproject.com

**Keywords:** Aboriginal food, insect honey, physicochemical properties, high-performance thin-layer chromatography, sugar content, antioxidant activity

## Abstract

Despite its cultural and nutritional importance for local Aboriginal people, the unusual insect honey produced by Western Australian honeypot ant (*Camponotus inflatus*) has to date been rarely investigated. This study reports on the honey’s physicochemical properties, its total phenolic, major sugars and 5-hydroxymethylfurfural contents, and its antioxidant activities. The honey’s color value is 467.63 mAU/63.39 mm Pfund, it has a pH of 3.85, and its electric conductivity is 449.71 µSiemens/cm. Its Brix value is 67.00, corresponding to a 33% moisture content. The total phenolics content is 19.62 mg gallic acid equivalent/100 g honey. Its antioxidant activity measured using the DPPH* (2,2-diphenyl-1-picrylhydrazyl) and FRAP (ferric reducing–antioxidant power) assays is 1367.67 µmol Trolox/kg and 3.52 mmol Fe^+2^/kg honey, respectively. Major sugars in the honey are glucose and fructose, with a fructose-to-glucose ratio of 0.85. Additionally, unidentified sugar was found in minor quantities.

## 1. Introduction

Honey is a sweet, viscous, and brown- to golden-colored natural product, which mainly consists of sugars (about 70–80% of the total solid), in particular monosaccharides (fructose and glucose, totaling approximately 75%) and minor quantities of disaccharides (e.g., sucrose) and polysaccharides [1,2]. It is produced by a variety of insects, but most commonly by the European honeybee (*Apis mellifera*), from the nectar of various plants. As nectar has a much higher moisture content (up to 93%) [3], honeybees actively evaporate off the water by fluttering their wings over the collected nectar, and they also add enzymes to break down the nectar sugars [4]. The end product of these activities is honey, which contains approximately 20% moisture, and due to its high sugar concentration, it is self-preserving. Next to sugars and residual water, honey also contains a range of enzymes, vitamin C, organic acids, phenolics, flavonoids, carotenoid derivatives, and amino acids, as well as proteins and minerals, which together make up approximately 3% of the total solid content of honey [5].

**Figure 1:**
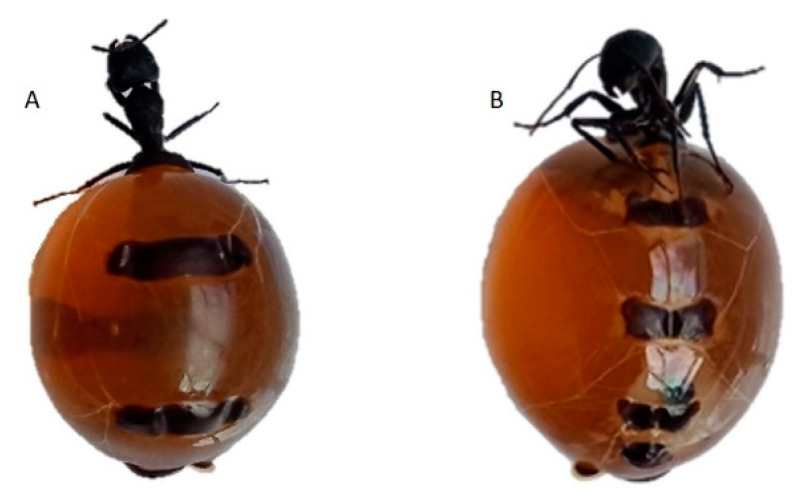
Australian honeypot ants’ posterior (dorsal) side view (**A**) and anterior (ventral) side view (**B**).

European honeybees (*Apis mellifera*) have been introduced throughout the world as the main producers of commercial honey [6]. However, honey can also be generated by other insects, including stingless bees, bumblebees, wasps, and so-called honeypot ants [7]. These unusual ants, which are found in dry, desert, or semi-arid regions [8], are reported from the USA (Colorado and New Mexico) [9], Mexico, the African continent, and also Australia [10,11]. In Australia, several species of honeypot ants have been recorded [12,13]; the most common is *Camponotus inflatus*. This particular species is closely associated with Mulga (*Acacia aneura*) trees, which provide shelter from high temperatures, act as a nectar source, and attract insect prey. The worker ants tend to feed on sap-sucking hemipterans living either on the phyllodes or roots of the Mulga trees. The ant colony itself lives underground, and amongst them, the “honeypot ants” are easily spotted, as these modified worker ants are over-fed by other worker ants to such an extent that their abdomens swell to the size of a small marble. Consequently, their abdomen wall is reduced to such a thin membrane that the honey stored within can be seen (Figure 1). These honeypot ants serve as a living food storage for the colony.

Honeypot ants play a significant role in the diet and culture of Australian Aboriginal people. Edie Ulrich, a Kutju woman from the Tjupan language group, comments: “Honey ants have been a part of the indigenous people’s diet for as long as we can remember. Legend has it that mothers who sit and gather the honey ants become so absorbed with digging that they forget to pay attention to their children and surroundings. This causes disaster as their enemies creep up to them unnoticed and slay their children before turning on them. All because of their love of digging for honey ants. This story has been passed down from generation to generation, reminding us ladies to be mindful of what is going on around us as we sit and gather.”

**Figure 2:**
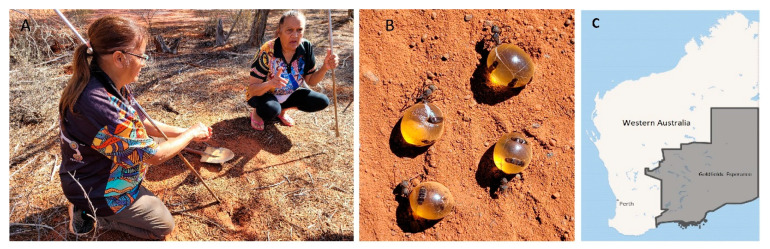
E. Ulrich and her sister Margorie Stubbs (**A**) collecting honeypot ants (**B**) about 50 km east of the city of Kalgoorlie in Western Australia’s Goldfields region (**C**).

To this day, not just Kultju people but many indigenous groups still collect honeypot ants. Digging for and gathering these ants is a woman’s job only (Figure 2). While men go off to hunt, women stay behind and dig for the ants, which is seen as a welcome opportunity for women to sit together, socialize, and yarn. It is also a great opportunity for children to enjoy and learn more about the bush, although they are told not to peer over the shoulders of the diggers into the hole because the honeypot ants will disappear. Locating the underground ant colonies is challenging and requires cultural and location-specific knowledge. Interestingly, it is believed that young children should not eat the honeypot ants because it will cause them to have problems with their speech (E. Ulrich, pers. comm.).

Given the visual and organoleptic similarities between honey derived from the activity of European honeybees and from the abdomen of honeypot ants, it is of interest to compare their chemical composition and antioxidant activity. Kinds of honey derived from honeybees have been extensively analyzed throughout the world for their chemical composition by a range of analytical techniques, including high-performance liquid chromatography–mass spectrometry (HPLC-MS), gas chromatography–mass spectrometry (GC-MS), nuclear magnetic resonance (NMR) spectrometry, near-infrared (NIR) spectroscopy, Raman spectroscopy, laser-induced breakdown spectroscopy, Fourier transform infrared (FT-IR) spectroscopy, plasma mass spectrometry, X-ray fluorescence spectroscopy, and high-performance thin-layer chromatography (HPTLC) [14,15,16,17], but only very few studies have to date been conducted on the honey produced by honeypot ants [18,19,20,21]. Badger and Korytnyk [18], in 1956, analyzed the sugar composition of Australian honeypot ants and found the major constituent sugar to be glucose and the fructose-to-glucose ratio to be 0.67. They also detected an unidentified disaccharide (other than sucrose) in the honey. However, any information on non-sugar constituents has, to our knowledge, to date not yet been published. Thus, there is a need to analyze in greater detail honey derived from Australian honeypot ants, specifically its physicochemical characteristics and antioxidant activity.

## 2. Results and Discussion

### 2.1. Appearance, Organoleptic and Physicochemical Parameters

The average weight of the ants was 1.26 ± 0.42 g (range 0.80–2.86 g). The ant’s abdomens were almost transparent, marble shaped (Figure 1 and Figure 2), and filled with a golden-brown liquid, referred to as “ant honey” throughout this paper.

According to the taste panelists, the ant honey was runnier with a less viscous consistency compared to the honeybee honey sample. The taste of the ant honey was described as sweet, but not as sweet as the comparator honeybee honey, and the ant honey was noted to have a sour undertone not detected in the comparator honeybee honey. The odor of the ant honey was described as typical of (honeybee) honey.

The ant honey had an average color reading of 467.63 mAU (SD = 7.33, *n* = 4). The mean Pfund value, which is sometimes used in the honey industry to describe a honey’s color, was found to be 63.39 mm (SD = 6.08, *n* = 4), which correlates to a light amber color. The color of honeybee honey is influenced by a variety of parameters, including its mineral and phenolics content, with darker honey being associated with a higher mineral and phenolics content [1]. A honey’s mineral content is influenced by its floral source or geographical origin and can be measured indirectly as electrical conductivity and ash content. The ant honey’s electrical conductivity was 449.71 µSiemens/cm (SD = 2.87), which is well below the limit of 0.8 mS/cm set by the Codex Alimentarius [22] for Honey published by the World Health Organization and the Food and Agriculture Organization of the United Nations. There was negligible residual ash upon incineration of the ant honey at 600 °C. The ant honey’s total phenolic content can be assumed, similar to what is seen for honeybee-derived honey, to be strongly influenced by its nectar source as well as the geographical and climatic characteristics of its harvest location [23]. It was determined as 19.62 mg gallic acid equivalent per 100 g of honey (SD = 0.38). Compared with the total phenolics data for a large range of Western Australian honeybee-derived types of honey (*n* = 451, unpublished data), which ranged from 7.39 to 75.56 mg gallic acid equivalent per 100 g of honey, the ant honey can be assessed as having a relatively low (9% percentile) total phenolics content. However, this conclusion is to be drawn with caution, as the total phenolics content is based on the total weight of the sample, which in the case of ant honey includes a relatively higher water content (see below), so a direct comparison of the total phenolics content cannot be made. The ant honey’s HPTLC derived organic extract fingerprint, which reflects its non-sugar composition, including many of its phenolic constituents, is shown in Section 2.5.

As the taste of the ant honey was slightly sour, which is mostly associated with acidic substances, the pH was measured and found to be 3.85 (SD = 0.04, *n* = 6). Typical pH values of honeybee-derived types of honey range between 3.2 and 4.5; thus, the pH of the ant honey falls within the expected range. As an acidic environment is seen as less conducive for microbial growth, the acidity of honey is considered as one factor (others being high osmolarity, the enzymatic generation of antibacterial hydrogen peroxide, as well as the presence of antibacterial constituents) that contributes to honey’s antibacterial activity. Future research should therefore explore the antibacterial activity of ant honey, which was outside the scope of this work.

The ant honey’s Brix value was 67.00 (SD = 0.20; *n* = 3); thus, the total sugar content is 67 g per 100 g of ant honey. Based on its Brix value, the honey’s moisture content can be determined to be 33%. This value is similar to the loss of moisture after 24 h freeze-drying, which was determined as 27.68% (SD = 0.74, *n* = 3). The ant honey seems to have a higher water content compared to typical honeybee-derived honey (not more than 20% according to the Codex Alimentarius [22] for Honey), which also explains its “runnier” and less viscous consistency. On return to the hive, honeybees disgorge the collected nectar into the honeycombs and then flutter their wings to evaporate some of the water, which transforms nectar into more viscous “honey” [24]. For ants, this process of removing water is not possible; hence, it is not surprising that ant honey has a higher moisture content compared to that produced by honeybees. Given the honeypot ants’ arid habitat, it can be speculated that ant honey not only serves as a source of nutrition for the colony but might also provide the ants feeding from the abdomen of the honeypot ants with water.

### 2.2. Total Antioxidant Activity

The total antioxidant activity of ant honey was measured using the DPPH* and FRAP assays. To ascertain the radical scavenging activity of the aqueous ant honey sample, the decay of the purple color of 2,2-diphenyl-1-picrylhydrazyl (DPPH) radicals in the presence of antioxidant compounds is measured with the change in absorbance being captured in a colorimetric assay at 517 nm. In the case of the ferric reducing-antioxidant power (FRAP) assay, the antioxidant activity is recorded in a spectrophotometric analysis as reduction of ferric 2,4,6-tris(2-pyridyl)-1,3,5-triazine [Fe (III)-TPTZ] to the corresponding ferrous complex at low pH. The antioxidant activity of ant honey was found to be 1367.67 µmol Trolox/kg (SD = 32.38) in the DPPH* and 3.52 mmol Fe^+2^/kg honey (SD = 0.19) in the FRAP assay, respectively. The antioxidant activity of a large range of Western Australian honeybee-derived honey samples (*n* = 451, unpublished data) ranged between 248.26 and 5407.76 µmol Trolox/kg in the DPPH* assay and between 0.97 and 11.66 mmol Fe^+2^/kg honey in the FRAP assay. In comparison with these findings, ant honey can be considered to possess moderate (DPPH—36% percentile and FRAP—32% percentile) antioxidant activity. However, a direct comparison is somewhat misleading given that in these assays, antioxidant activity is expressed with reference to the sample’s total weight and hence can be assumed to also be influenced by its moisture content. Given the higher water content of ant honey compared to typical honeybee-derived honey, lower activity data might thus not necessarily translate into a lower content of antioxidant honey constituents.

### 2.3. Sugar Analysis

**Figure 3:**
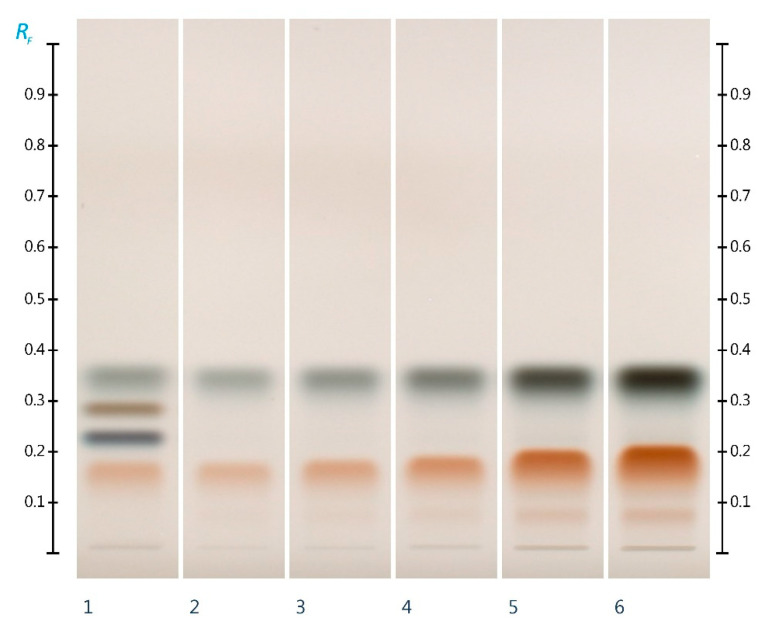
HPTLC images taken at white light after derivatization with aniline–diphenylamine–phosphoric acid reagent; Track 1—standards (fructose, maltose, sucrose, and glucose in increasing Rf values), Track 2—2 µL, Track 3—3 µL, Track 4—5 µL, Track 5—10 µL, and Track 6—15 µL of aqueous methanolic ant honey solution.

The main sugars present in the ant honey were qualitatively and quantitatively analyzed using HPTLC. Two different sample volumes (10 µL and 15 µL) were first applied in order to detect its predominant sugars. In this qualitative analysis step, it was found that next to glucose (Rf 0.33) and fructose (Rf 0.15), another sugar of yet unknown chemical identity is present in the sample (Rf 0.06) (Figure 3; Track 5 and 6). For quantification of the ant honey’s glucose and fructose content, 2 µL and 3 µL of the ant honey solution were analyzed. The two major sugars were quantified (Table 1), and the honey’s fructose to glucose ratio was found to be 0.85. This indicates that the ant honey is significantly different from honey produced by honeybees, where the fructose-to-glucose ratio is typically between 1 and 1.2. The findings of this study on the sugar composition of ant honey are in line with the early work by Wetherill (1853) and also by Badger and Korytnyk (1956) on ant honey; they found its major sugar to be glucose with a fructose-to-glucose ratio of 0.67 [18,19].

### 2.4. 5-Hydroxymethylfurfural (HMF) Analysis

**Figure 4:**
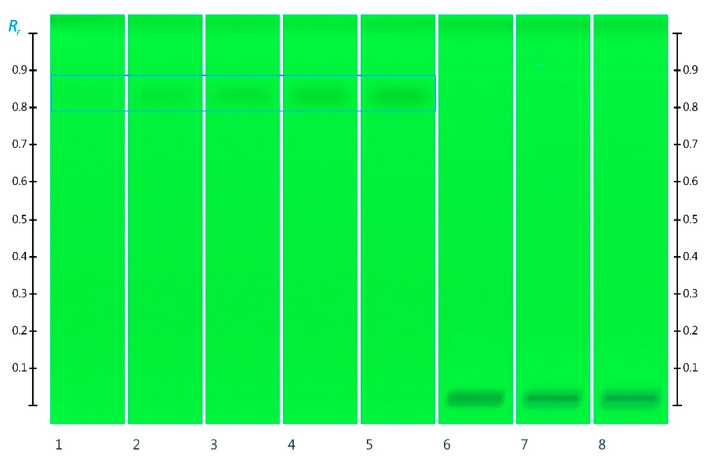
HPTLC images taken at 254 nm after development; Track 1, 2, 3, 4, and 5 were HMF (Rf 0.83) standard solution 1 µL, 2 µL, 3 µL, 4 µL, and 5 µL, respectively; Track 6, 7, and 8–10 µL aqueous ant honey solution.

HMF is formed through a Maillard reaction from reducing sugars in honey in an acidic environment at elevated temperatures [25,26]. It is considered a less desirable honey constituent since higher concentrations of HMF are associated with negative health implications [27]. The International Food Standards’ Codex Alimentarius, therefore, sets the maximum acceptable limit of HMF in honey at 40 mg/kg or at 80 mg/kg for types of honey produced in tropical regions (www.fao.org, accessed on 1 March 2022; Standard for Honey CXS 12-19811). As the Honeypot Ants live in a region classified as semi-arid, characterized by high temperatures throughout the day and cool nights (http://www.bom.gov.au/wa/kalgoorlie/ and climate-data.org, accessed on 1 March 2022; Climate Kalgoorlie-Australia), it can be anticipated that the honey they produce might be exposed to elevated temperatures and thus might contain HMF. The potential presence of HMF in ant honey was analyzed by HPTLC, but no quantifiable amounts of HMF were detected (Figure 4). This indicates that the honey stored inside the abdomen of honeypot ants living in underground nests is well protected from high temperatures and is not subject to an excessive Maillard reaction leading to HMF formation.

### 2.5. Organic Extract Analysis

**Figure 5:**
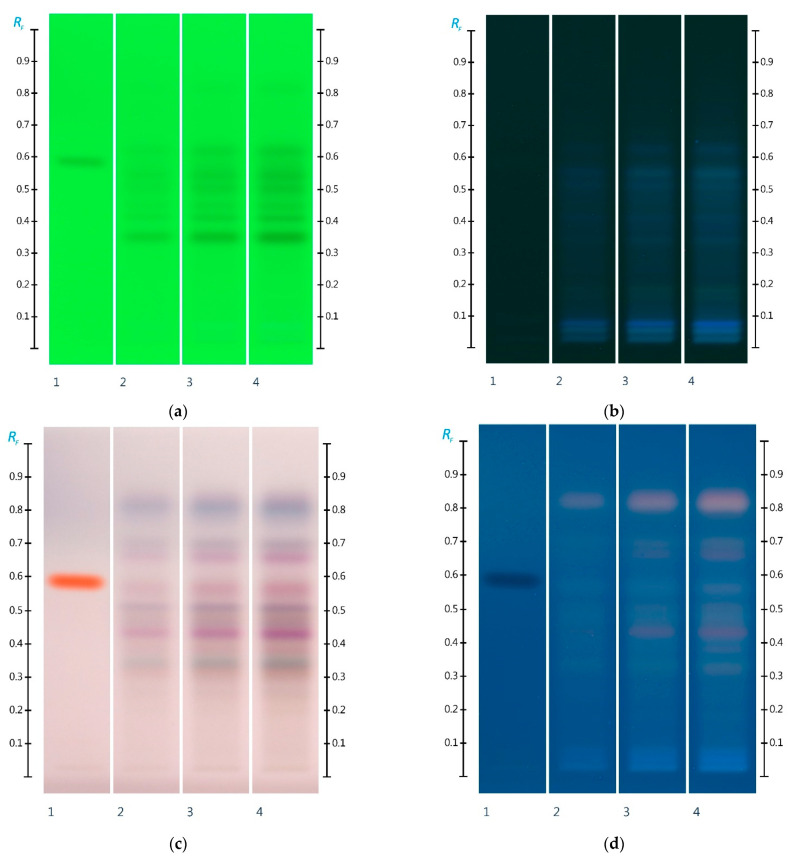
HPTLC images taken at (**a**) 254 nm, (**b**) 366 nm, (**c**) white light, and (**d**) 366 nm after derivatization with vanillin reagent; Track 1—4,5,7-trihydroxyflavanon, Track 2—5 μL, Track 3—10 μL, and Track 4—15 μL ant honey extract.

HPTLC fingerprint analysis was unable to detect any strong bands in the organic extract of the ant honey at lower concentrations (e.g., 5 µL application volume). At higher concentrations (e.g., 10 µL and 15 µL), major bands could be seen between Rf 0.30 and 0.85 indicating the presence of a range of (most likely phenolic) compounds. At R 254, bands were detected at Rf 0.35, 0.40, 0.45, 0.50, 0.54, and 0.62 (Figure 5a), and at R 366 after development, bands were detected at Rf 0.34, 0.41, 0.51, 0.55, and 0.63 (Figure 5b). At white light after derivatization, several bands could be detected at Rf 0.34 (orange), 0.35 (faint green) 0.42 (pink), 0.50 (ash), 0.56 (reddish), 0.66 (pink), 0.69 (dull purple), and 0.81 (blue) (Figure 5c). At R 366 after derivatization, bands were found at Rf 0.33, 0.43, 0.49, 0.58, 0.66, 0.69, and 0.81.

At this stage, the chemical identity of any of these compounds is unknown; it can, however, be assumed that they represent typical ant honey constituents, which are reflective of the honey’s specific collection site as well as the ants’ natural foraging sources. Future research should endeavor to identify some of these constituents, as they might be useful as chemical marker compounds of the ant honey (see Section 2.6).

### 2.6. HPTLC–DPPH Fingerprint Analysis

The total antioxidant activity as measured by the FRAP and DPPH assays does not identify individual active constituents. This makes it difficult to compare the antioxidant activity of different types of honey at a constituent level, as types of honey that yield the same total antioxidant activity may differ in the chemical composition of their active compounds [28]. Using HPTLC–DPPH fingerprint analysis (Islam et al., 2021), however, the antioxidant constituents of the ant honey’s organic extract are separated from each other during development. DPPH* is purple in color but, in its reduced state, turns into an orange-yellow color. Thus, the higher the antioxidant activity of a compound, the stronger the decolorization of the plate’s background color for the respective band. With this method, the quantification of the antioxidant activity of individual compounds is possible, even if their chemical identity is not yet known, by expressing their respective activity as gallic acid equivalents.

**Figure 6:**
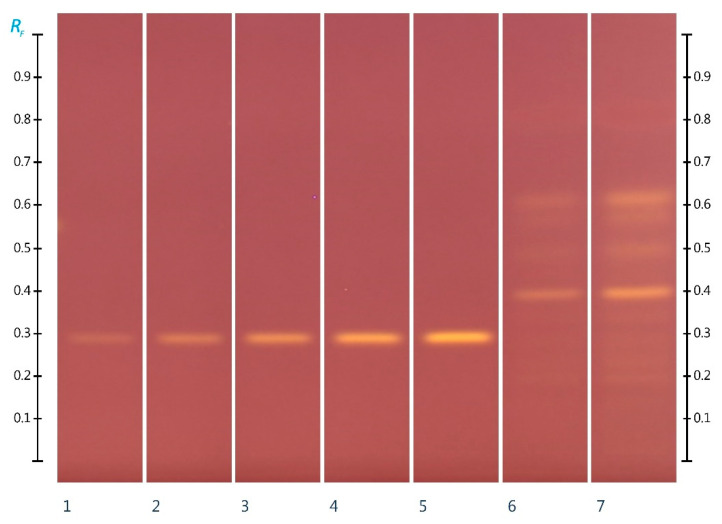
HPTLC images taken at transmission white (T white) light at 60 min after derivatization with 0.4% DPPH reagent; Track 1–5—Gallic acid (Rf 0.29) 2 µL, 3 µL, 4 µL, 5 µL, and 6 μL standards in methanol; Track 6—5 μL, and Track 7—10 μL ant honey extract.

The HPTLC–DPPH fingerprint analysis of the ant honey unveiled two major antioxidant bands (Rf 0.40 and 0.62) and six minor bands (Rf between 0.18 and 0.39 and between 0.50 and 0.59) (Figure 6). The total antioxidant band activity of these eight ant honey constituents was calculated as 0.24 mg equivalent gallic acid per 100 g ant honey. With this, the total band activity of antioxidant constituents in the ant honey’s organic extract is similar to that found for a honeybee-derived Western Australian multifloral honey and about a quarter of the total band activity recorded for an Australian Manuka honey [28].

## 3. Materials and Methods

### 3.1. Chemicals and Reagents

Chemicals and reagents, and their sources: Glucose, Sucrose, Sodium carbonate anhydrous (Chem-Supply Pty Ltd., St. Gillman, SA, Australia), Fructose, Maltose, Aniline, Vanillin (Sigma-Aldrich, St. Louis, MO, USA), Boric acid (Pharma Scope, Welshpool, WA, Australia), 4,5,7-trihydroxyflavanone and 5-hydroxymethylfurfural (Alfa Aesar, England, UK), DPPH* (Fluka AG, Buchs SG, Switzerland), Gallic acid, Diphenylamine, Phosphoric acid, 3,4,5-trihydroxybenzoic acid (Ajax Finechem Pvt Ltd., Sydney, NSW, Australia), Anhydrous sodium sulfate (Merck KGaA, Darmstadt, Germany), Folin and Ciocalteu’s Phenol Reagent 2N (Sigma-Aldrich, St. Louis, MO, USA).

Solvents and their sources: Methanol (Scharlau, Barcelona, Spain), 1-Butanol (Chem-Supply Pty Ltd., St. Gillman, SA, Australia), 2-Propanol (Asia Pacific Specialty Chemicals Ltd., Sydney, Australia), Dichloromethane (Merck KGaA, Darmstadt, Germany), Toluene (APS Chemicals, Sydney, NSW, Australia), Ethanol, Ethyl acetate and Formic acid (Ajax Finechem Pvt Ltd., Sydney, NSW, Australia).

### 3.2. Collection and Harvesting of Ant Honey

Twenty-three honeypot ants were collected in November 2020, 50 km east of Kalgoorlie in the Goldfields region in Western Australia. The countryside east of Kalgoorlie consists of large gumtrees that make up a part of the Great Western Woodlands. Amongst them, small pockets of Mulga (*Acacia aneura*) trees can be found where the honeypot ants were collected. The ant species was confirmed as Camponotus inflatus by staff from the School of Biological Sciences at the University of Western Australia. The ant honey was harvested by euthanizing the insects in a chloroform-filled chamber before squeezing the honey from their abdomen. The extracted ant honey was stored at 4 °C until further analysis.

### 3.3. Physicochemical Parameters

#### 3.3.1. Taste and Odor

A blind taste test comparing the ant honey with honeybee-derived honey from Western Australia was conducted by eight individuals. A generalized description of the ant honey’s taste and odor by the taste panelists was also recorded.

#### 3.3.2. Color and Pfund Value

Color was determined by dissolving the ant honey in deionized water to 50% (*w*/*v*) before measuring the optical density at 450 nm and 720 nm using a UV-Vis Spectrophotometer (Cary 60, Agilent Technologies, Santa Clara, CA, USA). The difference in the two optical density values was calculated, then multiplied by 1000, and expressed in milli-absorbance units (mAU) [29].

For the determination of Pfund value, 1 g of ant honey was dissolved in 2 mL of deionized water, and the absorbance of the ant honey solution was measured with a UV-Vis Spectrophotometer (Cary 60, Agilent Technologies, Santa Clara, CA, USA) at 635 nm [30]. The Pfund value was calculated as

Pfund [mm] = −38.7 + 371.39 × Abs

where Pfund = color value in Pfund scale, and Abs = absorbance at 635 nm.

#### 3.3.3. pH and Electrical Conductivity

The pH of the ant honey was measured by dissolving 1 g of honey in 7.5 mL of carbon-dioxide-free water; then, the pH was determined with a calibrated pH Meter (Eutech PC 2700-Eutech Instruments, Vernon Hills, Illinois, US) at room temperature [31].

A solution of 20% (*w*/*v*) ant honey was prepared in deionized water. The electrical conductivity of this solution was measured at 22 °C using an Electrical Conductometer (Eutech PC 2700-Eutech Instruments, Vernon Hills, Illinois, US), and the obtained data were expressed as microSiemens per centimeter (µS/cm) [32].

#### 3.3.4. Total Sugar

The Brix value of ant honey was measured using a Refractometer (HI96801, Hanna Instruments, Smithfield, RI, USA). A 40% (*w*/*w*) of sucrose solution was used for the calibration of the instrument. The obtained Brix value directly corresponds to the total sugar content, which was expressed in g per 100 g of honey.

#### 3.3.5. Water Content

The ant honey’s water content was determined using a Refractometer (HI96801, Hanna Instruments, Smithfield, RI, USA) and expressed as a percentage (*w*/*w*).

#### 3.3.6. Dry Matter after Freeze Drying and Ash Content

Ant honey at 1 g was put in a pre-weighed aluminum tray and freeze-dried overnight. After removal from the Freeze dryer (Alpha 1-2 LDplus, Martin Christ GmbH, Germany), the sample was weighed again to calculate its moisture loss. The freeze-dried ant honey was then placed in an electric furnace (ModuTemp furnace, Midvale, WA, Australia) at 600 °C for six hours followed by cooling in a desiccator, before weighing again to calculate its ash content, which was expressed as a percentage of its dry weight [33]:

Ash (%) = (W/W_0_) × 100

where W = weight of ash, and W_0_ = dry weight of honey

#### 3.3.7. Determination of Total Phenolics Content

The ant honey was warmed in a water bath set at no more than 40 °C prior to weighing to allow the honey to slightly liquefy to ensure homogenous sampling for weighing. The weighed honey was diluted (in triplicate) to 20% (*w*/*v*) with deionized water, followed by vortexing to ensure complete homogeneity.

An artificial honey solution was prepared by mixing 21.63 g of fructose, 18.13 g of glucose, 1.00 g of maltose, 0.75 g of sucrose, and 8.50 g of water. The sugar mixture at 2 g was diluted to 5 mL with deionized water.

Folin–Ciocalteu reagent at 1 mL was mixed with 30 mL deionized water. A 0.75% anhydrous sodium carbonate solution was prepared. Gallic acid standards ranging from 0.18 mg/mL to 0.06 mg/mL were prepared from a 2 mg/mL gallic acid stock solution.

Samples were prepared in triplicates as follows: (a) 200 µL of honey solution, (b) 100 µL of gallic acid standard spiked with 100 µL artificial honey, and (c) 100 µL water spiked with 100 µL artificial honey (blank). Each sample was mixed with 1 mL Folin–Ciucalteu reagent in a test tube and allowed to react for 5 min before 800 µL of Na_2_CO_3_ solution was added. The mixture was incubated in the dark for 2 h, and its absorbance was recorded at 760 nm (Cary 60 UV-Vis Spectrophotometer, Agilent Technologies, Santa Clara, CA, USA). The total phenolics content in each sample was expressed as mg of gallic acid equivalent per 100 g of ant honey [34].

#### 3.3.8. Total Antioxidant Activity

##### Ferric Reducing–Antioxidant Power (FRAP) Assay

Triplicate 20% (*w*/*v*) ant honey samples were prepared as described in Section 3.3.7. The FRAP reagent consisted of a 1:1:10 (*v*/*v*/*v*) ratio of 10 mM TPTZ (2,4,6-Tri(2-pyridyl)-s-triazine) (0.3123 g dissolved in 100 mL 40 mM HCl), 20 mM FeCl_3_·6H_2_O (0.5406 g dissolved in 100 mL deionized water) and 300 mM acetate buffer (pH 3.6; 3.1 g of sodium acetate, 16 mL of glacial acetic acid dissolved in 1000 mL deionized water). The reagent was prepared fresh and incubated at 37 °C prior to each assay [35].

Working standards of FeSO_4_·7H_2_O (from 200 to 1200 µM) were prepared from a 2 mM stock solution of FeSO_4_·7H_2_O (55.6 mg in 100 mL deionized water), stored on ice and used within two hours. The 600 µM standard served as positive control.

Ant honey samples and FeSO_4_·7H_2_O standards at 20 µL were each mixed with 180 µL of FRAP reagent in a Greiner 96-well flat-bottom microplate (Greiner Bio-One GmbH, Kremsmünster, Austria). The reaction mixtures were incubated at 37 °C, and their absorbance at 620 nm was determined after 30 min using a POLARstar Optima (BMG Labtech, Allmendgrün, Ortenberg, Germany) Microplate Reader. FRAP activity, expressed as mmol Fe*^+^*^2^ /kg fresh weight of ant honey, was derived from the interpolation of the standard curve as follows:

FRAP Value of Sample (µM)Fe (II) = (ΔAbs − intercept)/slope

##### 2,2-Diphenyl-1-picrylhydrazyl (DPPH) Assay

Triplicate 20% (*w*/*v*) ant honey samples were prepared as described in Section 3.3.7. Aqueous Trolox solutions (adjusted to pH 7.0) in a concentration range of 600–100 µM were used to derive the standard curve. The 400 µM Trolox standard served as positive control [36].

Ant honey samples and Trolox standards at 10 µL were placed in a Greiner 96-well flat-bottom microplate, followed by the addition of 100 µL of 100 mM of sodium acetate buffer (pH 5.5, 7.355 g of sodium acetate and 0.621 g of acetic acid dissolved in deionized water up to 1 L) and 290 µL of 0.130 mM of DPPH* reagent (5.1262 mg DPPH* in 100 mL methanol). The reaction mixture was incubated in the dark for 120 min before the absorbance was measured at 520 nm using the POLARstar Optima (BMG Labtech, Allmendgrün, Ortenberg, Germany) Microplate Reader. The mean radical scavenging activity of the ant honey sample was expressed as % DDPH* inhibition, calculated by linear regression analysis, and then expressed as µmol Trolox equivalent per kg of ant honey.

### 3.4. High-Performance Thin-Layer Chromatography (HPTLC) Analysis

#### 3.4.1. Standards, Reagent, and Mobile Phase Preparation

For the organic extract fingerprinting, a methanolic solution of 0.5 mg/mL of 4,5,7-trihydroxyflavanone was prepared as a reference standard. A mixture of toluene–ethyl acetate–formic acid (6:5:1, *v*/*v*/*v*) was used as the mobile phase. The vanillin derivatization reagent was prepared by dissolving 1 g of vanillin in 100 mL of ethanol followed by the dropwise addition of 2 mL of sulfuric acid. The antioxidant derivatization reagent was prepared by dissolving 40 mg DPPH* in 10 mL of a mixture of 50% methanol and 50% ethanol and stored in an amber glass bottle, protected from light, until use.

To identify and quantify the honey’s main sugars, standard glucose, fructose, sucrose, and maltose solutions (250 μg/mL) were prepared by dissolving 25 mg of the respective sugar in 100 mL of 50% aqueous methanol. A 3:5:1 mixture of 1-butanol–2-propanol–boric acid (5 mg/mL in water) was used as a mobile phase. The derivatization reagent was prepared by dissolving 2 g of diphenylamine and 2 mL of aniline in 80 mL of methanol. After the addition of 10 mL of phosphoric acid (85%), the solution was made up to 100 mL using methanol.

To detect and quantify the presence of 5-hydroxymethylfurfural, an aqueous 0.01% (*w*/*v*) solution of 5-hydroxymethylfurfural (HMF) was prepared as standard. A mixture of methanol and water (7:1, *v*/*v*) was used as a prewashing solvent for the HPTLC glass plates. Ethyl acetate was used as a mobile phase.

#### 3.4.2. Preparation of Samples for Analysis

For organic extraction, approximately 1 g of ant honey was mixed with 2 mL of deionized water. The aqueous solution was then extracted three times with 5 mL of dichloromethane. The combined organic extracts were dried with anhydrous MgSO_4_, filtered, and the solvent evaporated at ambient temperature. The extract was stored at 4 °C and reconstituted in 100 µL dichloromethane prior to HPTLC analysis.

For the sugar analysis, 100 mg of ant honey were dissolved in 80 mL of 50% aqueous methanol by sonication, then made up to 100 mL with 50% aqueous methanol.

A 10% (*w*/*v*) aqueous solution of ant honey was used for the analysis of its 5-hydroxymethylfurfural (HMF) content.

#### 3.4.3. Sugar Analysis

The standard solutions were applied as 8 mm bands at 8 mm from the lower edge of the HPTLC plate (glass plates 20 × 10 cm, silica gel 60 F_254_) at a rate of 50 nLs-1 using a semi-automated HPTLC application device (Linomat 5, CAMAG, Muttenz, Switzerland). To prepare the glucose, fructose, sucrose, and maltose standard curves 1 µL, 2 µL, 3 µL, 4 µL, and 5 µL of the respective standard solutions were applied. For the analysis of sugars in the ant honey, 3 µL of the sample solution was applied.

The chromatographic separation was performed in a saturated (33% relative humidity) automated development chamber (ADC2, CAMAG, Muttenz, Switzerland). The development chamber was saturated for 60 min, the plates were pre-conditioned with the mobile phase for 5 min, and automatically developed to a distance of 85 mm at fixed ambient temperature and dried for 5 min. The obtained chromatographic results were documented using a HPTLC imaging device (TLC Visualizer 2, CAMAG, Muttenz, Switzerland) under white light.

After initial documentation of the chromatographic results, each plate was derivatized with 2 mL of aniline–diphenylamine–phosphoric acid reagent using a TLC derivatizer (CAMAG Derivatiser, Muttenz, Switzerland). The derivatized plate was heated for 10 min at 115 °C using a TLC Plate Heater III (CAMAG, Muttenz, Switzerland). The plate was then cooled to room temperature and analyzed with the HPTLC imaging device under transmission white (T white) light [37]. The chromatographic images were digitally processed and analyzed using a specialized HPTLC software (visionCATS, CAMAG, Muttenz, Switzerland) which was also used to control the individual instrumentation modules.

#### 3.4.4. 5-Hydroxymethylfurfural (HMF) Analysis

The chromatographic separation was performed at ambient temperature on silica gel 60 F_254_ HPTLC plates (glass plates 20 × 10 cm). The HPTLC plates were prewashed with a mixture of methanol and water (7:1, *v*/*v*) and dried in an oven at 110 °C for 20 min.

The standard solutions were applied as 8 mm bands at 8 mm from the lower edge of the HPTLC plate at a rate of 50 nLs-1 using a semi-automated HPTLC application device (Linomat 5, CAMAG, Muttenz, Switzerland). To prepare the 5-hydroxymethylfurfural (HMF) standard curve, 1 µL, 2 µL, 3 µL, 4 µL, and 5 µL of the respective standard solution were applied. For the analysis of 5-hydroxymethylfurfural (HMF) in the ant honey, 10 µL of the sample solution was applied.

The following automated development chamber (ADC2, CAMAG, Muttenz, Switzerland) settings were used: pre-drying time 1 min, humidity control (33% relative humidity), tank saturation, and drying time 5 min. The plates were automatically developed to a distance of 50 mm at ambient temperature using ethyl acetate as a mobile phase. The obtained chromatographic results were documented using a TLC Scanner 4 (CAMAG, Muttenz, Switzerland) at 290 nm and HPTLC imaging device (TLC Visualizer 2, CAMAG, Muttenz, Switzerland) at 254 nm. The chromatographic results were analyzed using a specialized HPTLC software (visionCATS, CAMAG, Muttenz, Switzerland), which was also used to control the individual instrumentation modules [38].

#### 3.4.5. Organic Extract Analysis

The reference standard (4 µL) and the organic extract of ant honey (5 µL) were applied as 8 mm bands at 8 mm from the lower edge of the HPTLC plate (glass plates 20 × 10 cm, silica gel 60 F_254_) at a rate of 150 nLs-1 using a semi-automated HPTLC application device (Linomat 5, CAMAG, Muttenz, Switzerland). The chromatographic separation was performed in a saturated (33% relative humidity) automated development chamber (ADC2, CAMAG, Muttenz, Switzerland) and developed to a distance of 70 mm at a fixed ambient temperature. The obtained chromatographic results were documented using an HPTLC imaging device (TLC Visualizer 2, CAMAG, Muttenz, Switzerland) under 254 nm and 366 nm, respectively.

After initial documentation of the chromatographic results, each plate was derivatized with 3 mL of vanillin reagent and heated for 3 min at 115 °C using a TLC Plate Heater III (CAMAG, Muttenz, Switzerland). The plate was cooled to room temperature and analyzed with the HPTLC imaging device (TLC Visualizer 2, CAMAG, Muttenz, Switzerland) under white light and 366 nm [15,39]. The chromatographic images were digitally processed and analyzed using a specialized HPTLC software (visionCATS, CAMAG, Muttenz, Switzerland), which was also used to control the individual instrumentation modules.

#### 3.4.6. HPTLC–DPPH Fingerprint Analysis

For the quantification of antioxidant constituents in the honey’s organic extract as gallic acid equivalents 4 µL of the reference solution, 4 µL of the gallic acid standard solution, and 5 µL of the ant honey extract were applied as 8 mm bands at 8 mm from the lower edge of the HPTLC plate (glass plates 20 × 10 cm, silica gel 60 F_254_) at a rate of 150 nLs-1 using a semi-automated HPTLC application device (Linomat 5, CAMAG, Muttenz, Switzerland). To prepare a gallic acid standard curve in the ant honey matrix, 2 µL, 3 µL, 4 µL, 5 µL, 6 µL, and 7 µL of gallic acid standard solution were applied by over-spotting the ant honey bands.

The chromatographic separation was performed in a saturated (33% relative humidity) automated development chamber (ADC2, CAMAG, Muttenz, Switzerland), developed to a distance of 70 mm at fixed ambient temperature, and dried for 5 min. The plate was derivatized with 3 mL of 0.4% DPPH* reagent (CAMAG derivatizer). The derivatized plate was analyzed with the HPTLC imaging device (TLC Visualizer 2, CAMAG, Muttenz, Switzerland) under white light by taking images 60 min after derivatization [40]. The obtained chromatographic images were digitally processed and analyzed using a specialized HPTLC software (visionCATS, CAMAG, Muttenz, Switzerland), which was also used to control the individual instrumentation modules. For the quantification of antioxidant honey constituents as gallic acid equivalents, the obtained images were converted into individual absorbance points according to their Rf values. Using Excel©, the data were converted into chromatograms, which were used to derive calibration curves of the area of absorbance vs. concentration.

## 4. Conclusions

Although honey produced by Australian honeypot ants (Camponotus inflatus) has been used by indigenous Aboriginal Australians for thousands of years, an in-depth analysis of this insect honey has not been reported. Its cultural significance and its value as an indigenous food source warrant an investigation of the honey’s physicochemical and antioxidant properties and, where possible, a comparison with honey derived from European honeybees. To our knowledge, this study is the first in-depth analysis of Australian honey produced by insects other than honeybees. It has found ant honey to have organoleptic characteristics similar to typical honeybee-derived honey, although it appears less sweet and runnier. Its acidic pH also falls into the typical pH range of honeybee-derived honey, but the ant honey’s moisture content is higher, which explains its runnier appearance. The ant honey contains a range of as yet not chemically identified non-sugar constituents that contribute to its antioxidant activity, which has been found to be moderate compared to honeybee-derived honey, although a direct comparison is difficult given the ant honey’s higher water content. A noticeable difference has also been found with respect to the ant honey’s main sugars, where glucose is present in higher quantities than fructose, which is opposite to honeybee-derived types of honey.

## Figures and Tables

**Table 1:** Glucose and fructose content and fructose-to-glucose ratio (F: G) of honey.

	Major Sugars						
	Glucose			Fructose			Ratio (F:G)
	Measured Content (ng/µL)	Mean	SD	Measured Content (ng/µL)	Mean	SD	
Run 1	371.60			315.10			0.85
Run 2	352.67	364.42	10.26	302.73	310.73	6.94	0.85
Run 3	369.00			314.37			0.85

## Data Availability

Not applicable.

## Sample Availability

Samples of the compounds are not available from the authors.
